# Melatonin increases collagen content accumulation and Fibroblast Growth Factor-2 secretion in cultured human cardiac fibroblasts

**DOI:** 10.1007/s43440-023-00490-4

**Published:** 2023-05-15

**Authors:** Marta Drobnik, Agnieszka Tomaszewska, Joanna Ryżko, Aleksandra Kędzia, Małgorzata Gałdyszyńska, Lucyna Piera, Justyna Rydel, Jacek Szymański, Jacek Drobnik

**Affiliations:** 1 Laboratory of Connective Tissue Metabolism, Department of Pathophysiology, Medical University of Lodz, Zeligowskiego 7/9, 90-752, Lodz, Poland; 2 Central Scientific Laboratory, Medical University of Lodz, Ul. Mazowiecka 6/8, 92-215 Lodz, Poland

**Keywords:** Cell cultures, Collagen, Connective tissue, Fibroblast, Glycosaminoglycan, Heart, Melatonin, Proliferation

## Abstract

**Background:**

The extracellular matrix serves as a scaffold for cardiomyocytes, allowing them to work in accord. In rats, collagen metabolism within a myocardial infarction scar is regulated by melatonin. The present study determines whether melatonin influences matrix metabolism within human cardiac fibroblast cultures and examines the underlying mechanism.

**Methods:**

The experiments were performed on cultures of cardiac fibroblasts. The Woessner method, 1,9-dimethylmethylene blue assay, enzyme-linked immunosorbent assay and quantitative PCR were used in the study.

**Results:**

Melatonin treatment lowered the total cell count within the culture, elevated necrotic and apoptotic cell count as well as augmented cardiac fibroblast proliferation, and increased total, intracellular, and extracellular collagen within the fibroblast culture; it also elevated type III procollagen α1 chain expression, without increasing procollagen type I mRNA production. The pineal hormone did not influence matrix metalloproteinase-2 (MMP-2) release or glycosaminoglycan accumulation by cardiac fibroblasts. Melatonin increased the release of Fibroblast Growth Factor-2 (FGF-2) by human cardiac fibroblasts, but cardiotrophin release was not influenced.

**Conclusion:**

Within human cardiac fibroblast culture, collagen metabolism is regulated by melatonin. The profibrotic effect of melatonin depends on the elevation of procollagen type III gene expression, and this could be modified by FGF-2. Two parallel processes, viz., cell elimination and proliferation, induced by melatonin, lead to excessive replacement of cardiac fibroblasts.

## Introduction

The extracellular matrix (ECM) forms the three-dimensional structure within the heart. It comprises fibrous proteins (collagen, elastin) and adhesive molecules such as fibronectin or laminin. The main proteins within the myocardium are type I and III collagen; these serve as a scaffold stabilizing the cells, as well as connect the cardiomyocytes allowing them to work as a pump. In addition, the ECM contains polysaccharides, or rather glycosaminoglycans (GAG). GAG and proteoglycans are associated with collagen-forming complexes [1]. GAG may also modify collagen fibrillogenesis in vitro [2]. GAG (except hyaluronate and keratan sulfate) may bind to collagen by electrostatic interactions at physiological pH and ionic strength [3]. The connective tissue also influences cell migration, spreading, and proliferation.

The most numerous cells involved in regulating ECM homeostasis within the heart are fibroblasts. They also detect physical stimuli, control cytokine release, and are involved in the stimulation or inhibition of angiogenesis [4, 5]. Although fibroblasts are not excitable cells, they contribute to the electrophysiological phenomena generated within the heart [4]. They also produce matrix metalloproteinases: the enzymes catabolizing collagen. Cardiac fibroblasts release cytokines such as Fibroblast Growth Factor-2 (FGF-2) and cardiotrophin. FGF-2 increases cardiac hypertrophy and influences ECM accumulation [6–8]. Cardiotrophin, a molecule belonging to the interleukin-6 superfamily, is responsible for the induction of heart fibrosis [9].

Melatonin is a hormone released by the pineal gland. It was defined by Reiter as the chemical expression of darkness because melatonin is released at night and light terminates their secretion [10]. Melatonin could be also synthesized in the intestine [11], the Harderian gland [12], skin [13], bone marrow [14], endothelial cells [12], and various other organs [12]. It influences the endocrine [15], nervous [16], and immune [17] systems. As a regulator of regulators, melatonin is believed to influence the homeostasis of the entire body. It may also protect the heart from the effects of ischemia/reperfusion injury [18]. In addition, experiments have shown a reduction of collagen content within the scar of myocardial infarction in rats [19]. Melatonin also exerts a regulatory influence on collagen metabolism and fibrosis of various organs. The final effect of melatonin was dependent on the applied dose, targeted organ, model of experiment, and species of the experimental subject. For example, physiological doses of the hormone increased collagen content in a myocardial infarction scar [20] but decreased collagen capacity within wound granulation tissue in rats [21]. Pharmacological doses of melatonin did not influence collagen levels within the myocardial infarction scar in rats [19].

Several studies in rats indicate that melatonin is involved in regulating collagen metabolism and fibrosis of organs [19–21]. Therefore, the present study aims to confirm whether melatonin is involved in regulating collagen metabolism in human cardiac fibroblast cultures and to determine its mechanism of action. In addition, the most important determinants of collagen metabolism, i.e., synthesis and catabolism are also investigated.

## Materials and methods

### Melatonin preparation

Melatonin (Sigma, St. Louis, USA, M5250) was dissolved in Dimethyl sulfoxide (DMSO, Sigma, St. Louis, USA, D2650) and then diluted with a culture medium. It was used at a concentration of 1 µM and 0.1 µM. Melatonin exerted more pronounced effects at the lower concentration (0.1 mM), than at the higher dose (1 mM). The DMSO solvent was applied at a final concentration of 0.001%.

### Cell cultures

An immortalized human cardiac fibroblast cell line was used (ABM, Richmond, BC, Canada, T044i6). The medium consisted of DMEM (Biowest,  Nuaillé, France, L0103) containing 3% fetal bovine serum (Biowest,  Nuaillé, France, S181H), amphotericin B (2.5 μg/ml), gentamicin (25 μg/ml), 50 µg/ml vitamin C (Sigma Aldrich, St. Louis, MO, USA, A4544) and 5 µg/ml insulin (Thermo Fisher Scientific, Waltham, MA, USA, 12585-014). The cells were cultured at 37 °C, 100% humidity in a mixture of 95% air and 5% CO_2_. The cardiac fibroblasts were cultured in 24-well and 96-well plates at initial cell densities of 5 × 10^4^/well or 2.5 × 10^3^/well, respectively. Both total and necrotic cells (stained with trypan blue) were counted in a Bürker chamber. The following experimental groups of cells were used: intact controls (CTR), cells administered with 0.001% DMSO, fibroblasts treated with melatonin at 1 µM (MLT-1) or 0.1 µM (MLT-0.1) with 0.001% DMSO.

### Quantitative PCR

The total RNA of the experimental fibroblasts was isolated using a minicolumn Total RNA Mini Kit (A&A Biotechnology, Gdynia, Poland, 031–100). The concentration of the isolated RNA was evaluated with a NanoDrop™ One Spectrophotometer (Thermo Fisher Scientific, Waltham, MA, USA).

RNA was transcribed into cDNA using a PrimeScript RT-PCR Kit (Takara, Kusatsu, Shiga, Japan) with 500 ng of RNA. The gene expression of α1 chain of type I and III procollagen, *RPLP13* (60S ribosomal protein L13a), and *YWHAZ (*tyrosine 3-monooxygenase/tryptophan 5-monooxygenase activation protein zeta) was measured with the Universal Probe Library (UPL) (Roche, Indianapolis, IN, USA). *GAPDH* (glyceraldehyde 3-phosphate dehydrogenase) expression was determined using Real-Time ready Custom Single Assay (Roche, Indianapolis, IN, USA).

*YWHAZ* gene expression was measured using the primers AAGTGCAATGGAGACCTTGG and GTTGCCCTAGATGCAGAAGG with UPL probe #2, while *RPLP13* expression was measured with GGCACCATTGAAATCCTGAG and GAAGGGGGAGATGTTGAGC and UPL probe #36.

The reaction was prepared using the FastStart Essential Probe Master (Roche, Indianapolis, IN, USA, 06 924 492 001), according to the manufacturer’s instructions. The reaction consisted of 10 min at 95 °C followed by 55 cycles of 10 s at 95 °C, 30 s at 60 °C and 1 s at 72 °C. The final step comprised a 30 s reaction at 40 °C. The relative expression of the investigated genes was calculated using LightCycler^®^ 96 software (Roche, Indianapolis, IN, USA). Each assessment was repeated twice.

### Enzyme-linked immunosorbent assay (ELISA)

The concentrations of matrix metalloproteinase-2 (MMP-2), FGF-2, and cardiotrofin were measured within the culture medium using ELISA kits (E-EL-H1445, E-EL-H6042 Elabscience, Wuhan, China, or QY-E05593 Qayee-bio Shan-ghai China, respectively). The measurements were performed according to the manufacturer’s protocol. Absorbance was determined at 450 nm using an EL × 800UV Universal Microplate Reader (BioTek Instruments Inc., Winooski, VT, USA).

### Collagen evaluation

Collagen content was evaluated using the Woessner method, as described previously [5]. After drying at 60 °C, each sample was hydrolyzed for 24 h at 110 °C with 6 N HCl. All hydrolysates were evaporated to dryness. The precipitate was then dissolved with 3 ml of redistilled water, and the acidic pH of each sample was neutralized using 1 N NaOH. All solutions were diluted to 10 ml with redistilled water. After further dilution of the tested sample with redistilled water (0.5 ml to 2 ml of the final volume), hydroxyproline was oxidized for 20 min at 20 °C to pyrrole using 1.25 ml chloramine T in a citrate buffer (pH = 6). To remove the chloramine T, 1.25 ml of 3.15 M perchloric acid was added and the samples were subjected to 20 min incubation at 60 °C with 1 ml of 20% p-dimethylaminobenzaldehyde. Optical density was measured at 560 nm using a spectrophotometer.

### Determination of GAG

GAG were evaluated by 1,9-dimethylmethylene blue (DMMB) assay, as described earlier [19]. After homogenization, all samples were dried at 60 °C. Following this, 50 mg samples were incubated for 1 h at 73 °C with a solution consisting of 0.75 M NaOH and 50 mM natrium borate. Then samples were neutralized to pH 7.0 using 6 M HCl. To precipitate the proteins, 100% TCA was added (72 μl/sample). The samples were centrifuged at 6000 rpm for 30 min and the supernatant was dissolved in 6 ml of 100% ethanol. The samples were stored in a refrigerator at − 20 °C overnight and then centrifuged (12 500 rpm for 30 min). The supernatant containing the GAG precipitate was dissolved in distilled water.

Each sample (50 μl) was added to 1.2 ml of DMMB-reagent (Aldrich Chemical Co), consisting of 51 mM DMMB, 45 mM glycine, and 41 mM NaCl. The pH was adjusted to 3.0 with 1 M HCl. The absorbance of each sample was evaluated at 525 nm with a spectrophotometer.

### Apoptosis analysis

Apoptosis was measured using an Annexin-FITC Apoptosis detection kit. The analysis was performed according to the manufacturer’s protocol. All samples were analyzed using a FACS Canto II Analytical Flow Cytometer (BDE Biosciences). The results were plotted using Kaluza Analysis version 1.5a (Beckman Coulter, Inc).

### Statistical analysis

The experimental data were analyzed using Statistica 13.1 software (StatSoft, Tulsa, OK, USA). The normality of the data was calculated with the Shapiro–Wilk test and the homogeneity of variance with Levene’s test. For the data that did not follow a normal distribution, or the variance was not homogenous, the Kruskal–Wallis’s test was used, with differences between the groups being evaluated with the Mann–Whitney *U* test. When the data followed a normal distribution and homogenous variance, a one-way ANOVA was used with Shaffer’s post hoc test. The minimal level of significance was *p* < 0.05.

## Results

### Fibroblasts

**Fig. 1:**
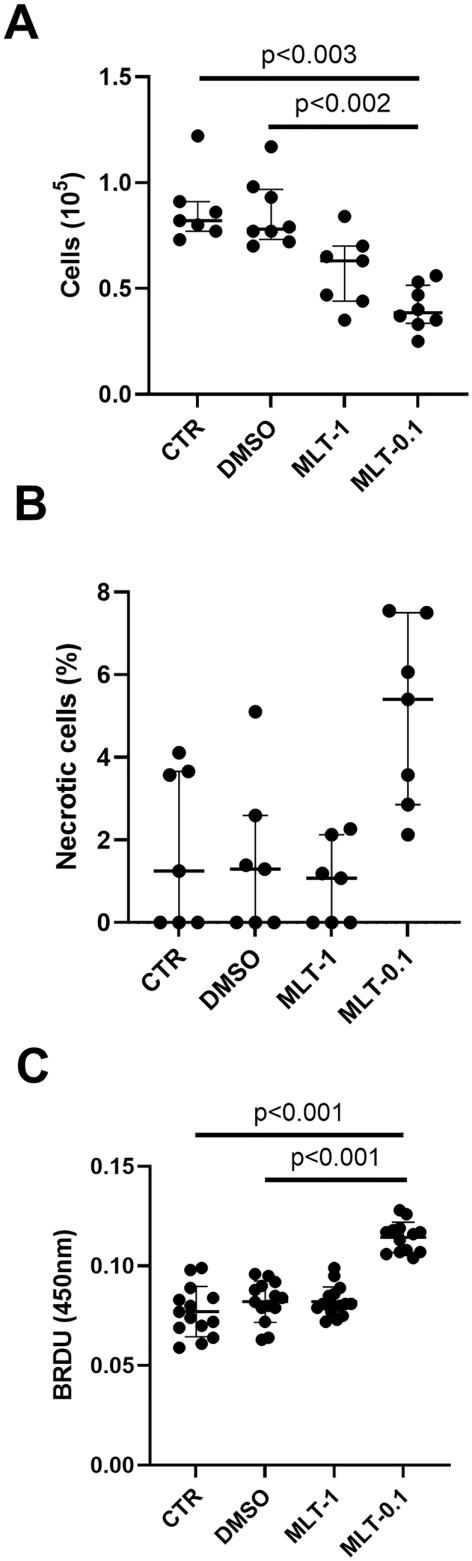
Total cell number counted in Bürker chamber (**A**), necrotic cell count recognized by trypan blue elimination assay (**B**), and fibroblasts proliferation measured by BrdU method (**C**) in controls (CTR) DMSO-treated cultures (DMSO) and fibroblasts administered with melatonin at the concentrations of 1 µM (MLT-1) and 0.1 µM (MLT-0.1). In **A** and **B**, values represent medians ± interquartile ranges (*n* = 7–8). Kruskal–Wallis’s test followed by Mann–Whitney *U* test; In **C**, each value represents mean ± SD. One-way ANOVA followed by Shaffer’s post hoc test

The controls (CTR) and DMSO-treated groups had similar total cell counts. Melatonin did not significantly decrease the total cell number within the culture when applied at a concentration of 1 µM, indicating no significant difference between the groups. However, when applied at a concentration of 0.1 µM, melatonin decreased total cell count within the culture compared with the control group (*p* < 0.003) and DMSO-administered cultures (*p* < 0.002, Fig. 1A) (Kruskal–Wallis test, H(3) = 8.179, *p* = 0.0005). In addition, the three groups demonstrated similar necrotic cell counts, stained with trypan blue, when melatonin was administered at 1 µM (MLT-1); however, 0.1 µM melatonin treatment insignificantly increased necrotic cell numbers (Kruskal–Wallis test H(3) = 7.30, *p* = 0.0628, MLT-0.1, Fig. 1B).

Regarding the effect of melatonin on cell proliferation, the controls (CTR), DMSO cultures, and cells treated with melatonin 1 µM (MLT-1) demonstrated similar levels of cardiac fibroblast proliferation, measured as the incorporation of bromodeoxyuridine. However, 0.1 µM melatonin (MLT-0.1) increased cell division compared to the control (one-way ANOVA, F_3.53_ = 41.79), followed by Scheffe test, CTR; *p* < 0.001) and DMSO groups (*p* < 0.001, Fig. 1C).

**Fig. 2:**
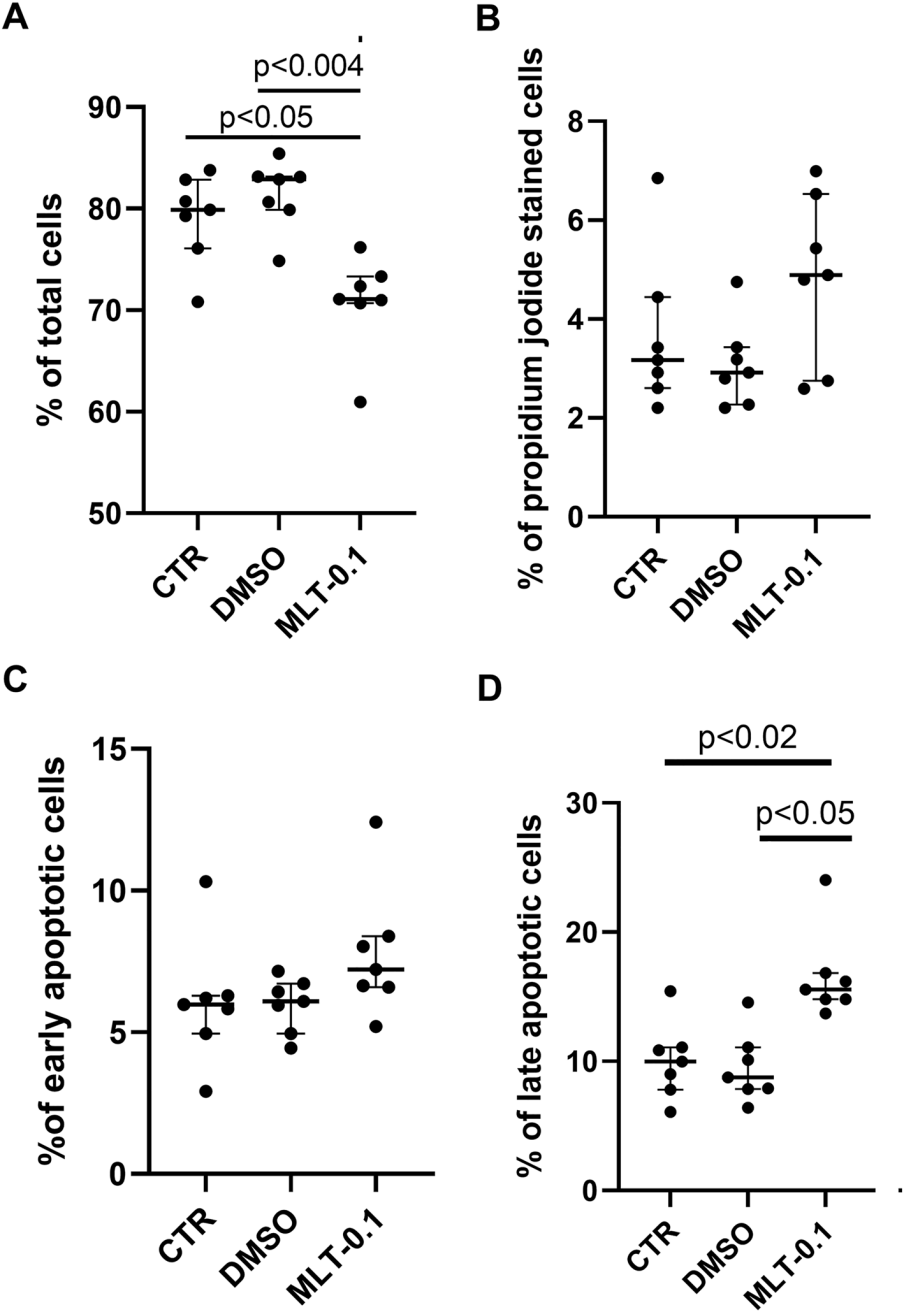
Total cell number (**A**), necrotic cell count (propidium iodide fibroblasts; (**B**) early (**C**) and late (**D**) apoptotic cell number in controls (CTR), DMSO-treated cultures (DMSO) and fibroblasts administered with melatonin at the concentrations 0.1 µM (MLT-0.1). All measurements were performed using an Annexin-FITC Apoptosis detection kit. Values represent medians ± interquartile ranges (*n* = 6–7). Kruskal–Wallis’s test followed by Mann–Whitney *U* test

Melatonin decreased the total cell number compared with the DMSO-treated cultures (*p* < 0.004) and the control cells (*p* < 0.05) (Kruskal–Wallis test, H(2) = 10.80, *p* = 0.0045). The controls and DMSO variants had identical total cell counts (Fig. 2A), and both demonstrated lower proportions of propidium iodide-stained, i.e., necrotic cells, compared with those receiving melatonin treatment (Fig. 2B) (Kruskal–Wallis test, H(2) = 3.47, *p* = 0.1757). Furthermore, melatonin treatment (0.1 µM) tended to increase early apoptotic cell count (Annexin V positive) compared with DMSO-treated cells and controls (Fig. 2C) (Kruskal–Wallis test, H(2) = 5.82, *p* = 0.543); it also increased the number of late apoptotic cells (propidium iodide and annexin V positive) compared to the DMSO group (*p* < 0.05) and control (*p* < 0.02, Fig. 2D) (Kruskal–Wallis test, H(2) = 11.32, p = 0.0035).

### Collagen

**Fig. 3:**
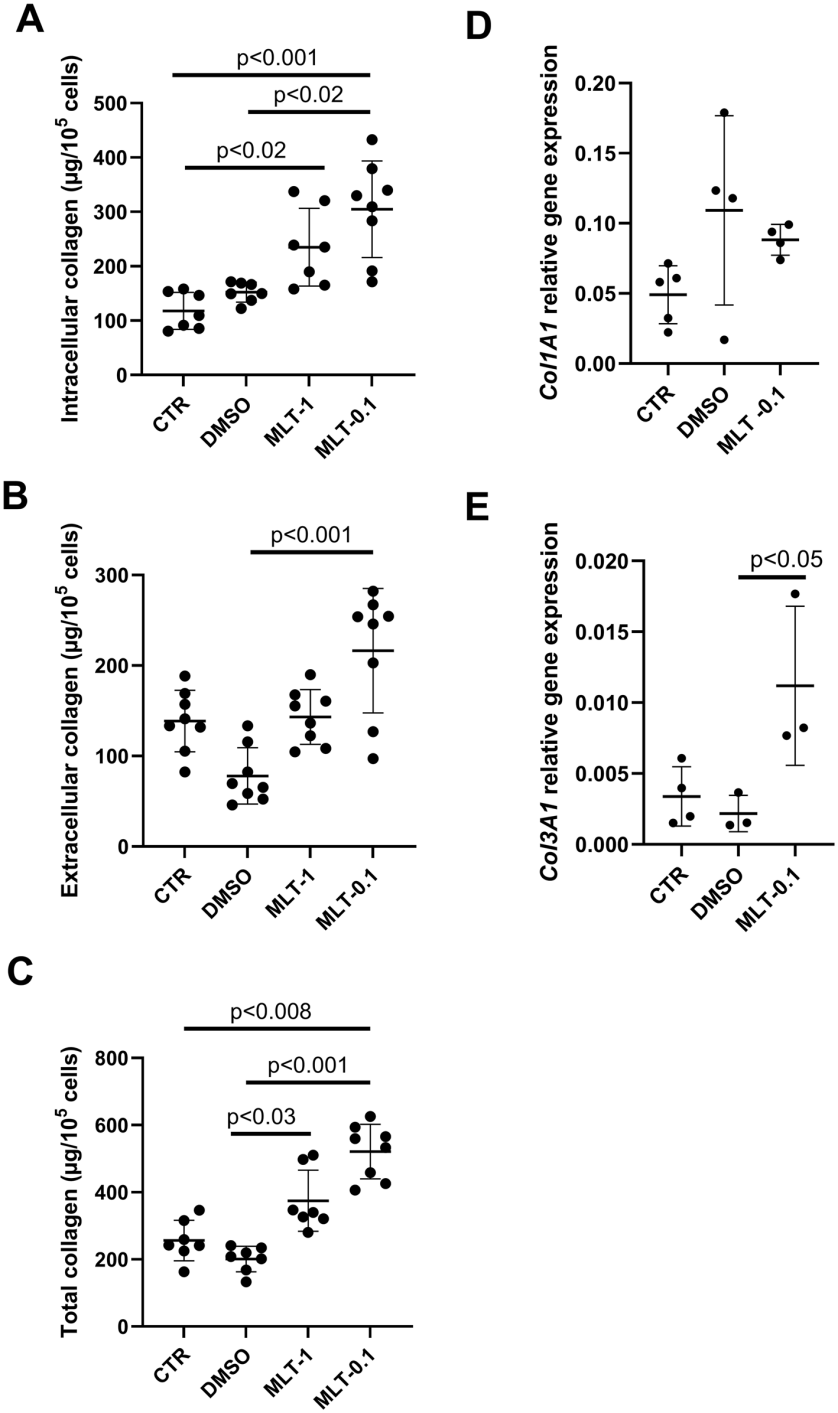
Intracellular collagen (**A**), extracellular collagen (**B**), and total collagen (**C**) content within controls (CTR), DMSO-treated cultures (DMSO), and fibroblasts administered with melatonin at the concentration of 1 µM (MLT-1) and 0.1 µM (MLT-0.1). Collagen was evaluated by the Woessner method. *Col1A1* (**D**) and *Col3A1* (**E**) gene relative expression were measured by real-time PCR in controls (CTR) DMSO-treated cultures (DMSO) and fibroblasts administered with melatonin at the concentration of 0.1 µM (MLT-0.1). Values represent medians ± interquartile ranges; *n* = 7–8 (**A**, **B**, **C**); *n* = 3–5 (**D**, **E**). Kruskal–Wallis’s test followed by Mann–Whitney *U* test

The intracellular collagen content of the two controls was identical. However, treatment with 1 µM melatonin (MLT-1) elevated collagen content compared with controls (CTR; *p* < 0.02), and 0.1 µM M treatment increased content compared to both untreated (CTR; *p* < 0.001) and DMSO controls (*p* < 0.02; Fig. 3A) (Kruskal–Wallis test, H(3) = 20.23, *p* = 0.0002).

Extracellular collagen content did not significantly differ between control groups. In addition, 1 µM melatonin did not change collagen level compared to control (CTR) and DMSO-treated cultures. However 0.1 µM melatonin significantly augmented collagen content compared with the DMSO-treated cells (*p* < 0.001, Fig. 3B) (Kruskal–Wallis test, H(3) = 16.11, *p* = 0.0011). No significant difference in total collagen content was found between controls (CTR) and DMSO controls. However, 1 µM melatonin significantly increased collagen level compared to the DMSO group (*p* < 0.03), and 0.1 µM increased the level compared with DMSO-treated cells (*p* < 0.001) and untreated controls (CTR; *p* < 0.008; Fig. 3C) (Kruskal–Wallis test, H(3) = 22.71, *p* = 0.0000).

Melatonin treatment (0.1 µM) did not change the procollagen type I α1 chain gene expression compared with the CTR and DMSO groups. The gene expression did not significantly differ between the controls (CTR) and DMSO-treated cultures (Fig. 3D) (Kruskal–Wallis test, H(2) = 2.34, *p* = 0.31). However, 0.1 µM melatonin increased procollagen type III α 1 chain gene expression compared to the DMSO-treated group (*p* < 0.05). Similar procollagen type III α 1 chain gene expression was observed between the controls and the DMSO group (Fig. 3E).

### GAG, cytokines, and matrix metalloproteinase-2

**Fig. 4:**
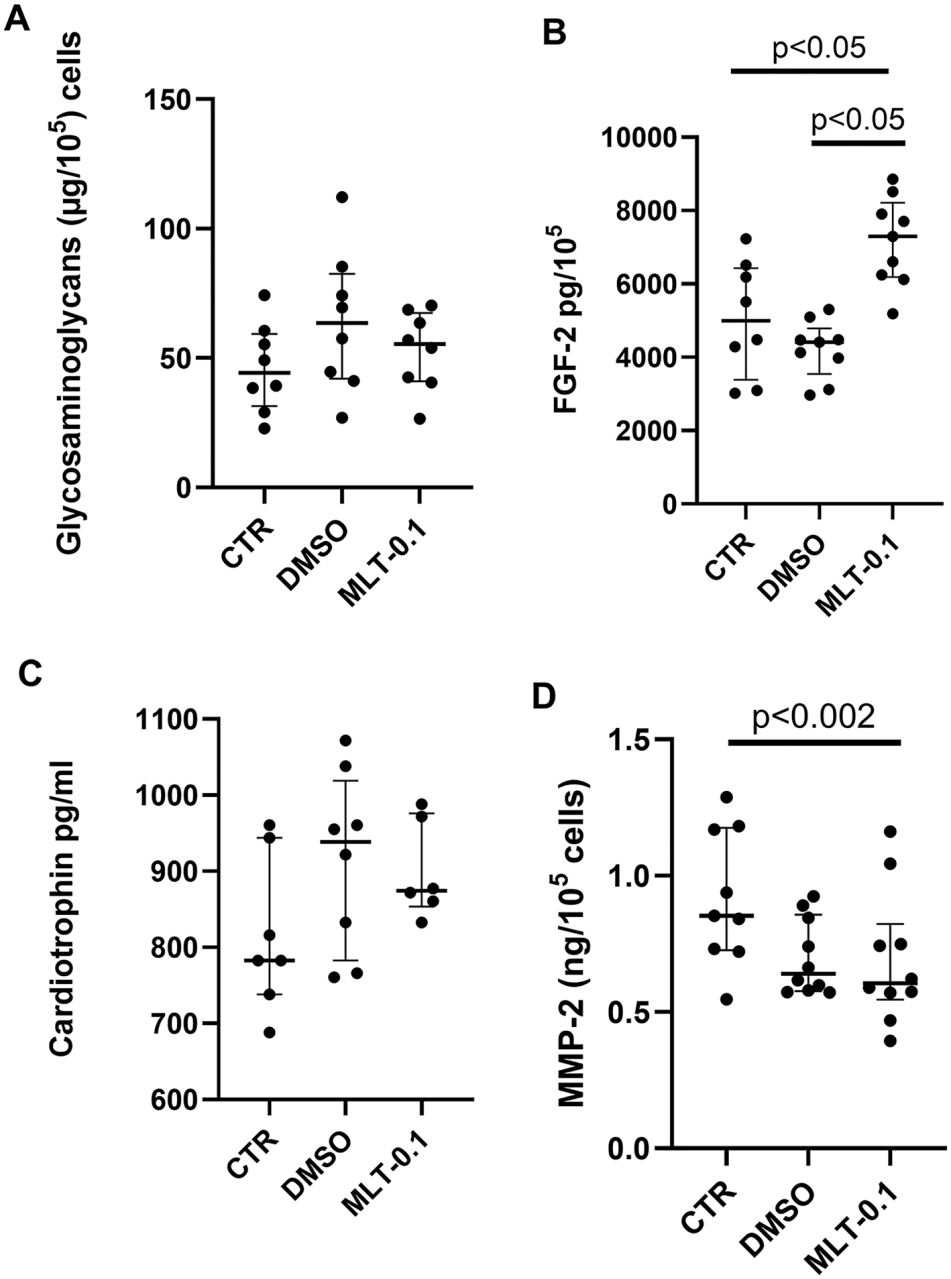
Glycosaminoglycan content (**A**) evaluated by 1,9-dimethylmethylene blue assay, within controls (CTR) DMSO-treated cultures (DMSO) and fibroblasts administered with melatonin at the concentration of 0.1 µM (MLT-0.1). FGF-2 (**B**), cardiotrophin (**C**), and MMP-2 (**D**) content evaluated by ELISA, in controls (CTR), DMSO-treated cultures (DMSO), and fibroblasts administered with melatonin at the concentration of 0.1 µM (MLT-0.1). Values represent medians ± interquartile ranges (*n* = 7–10). Kruskal–Wallis’s test followed by Mann–Whitney *U* test

Similar levels of GAG were found in all tested groups (Fig. 4A). Similar secretion FGF-2 was identified in both control (CTR) and DMSO-treated cultures. However, augmented FGF-2 secretion was observed in the MLT-0.1 group (0.1 µM) compared with controls (CTR; *p* < 0.05) and the DMSO group (*p* < 0.05, Fig. 4B) (Kruskal–Wallis test, H(2) = 13.74, *p* = 0.001).

Melatonin treatment (MLT-0.1, 0.1 µM) did not influence cardiotrophin release (Fig. 4C) or MMP-2 release by cardiac fibroblasts. Lower MMP-2 release was observed in the DMSO group compared to the untreated controls (CTR; *p* < 0.02). Melatonin treatment reduced MMP-2 levels compared to untreated controls (CTR), but not compared to the DMSO-treated controls (Fig. 4D).

## Discussion

Our findings confirm that melatonin has a regulatory effect on collagen accumulation in human cardiac fibroblast cultures, with treatment increasing total, intracellular, and extracellular collagen content, thus appearing to support fibrotic processes in this organ. The present data comprise the first such observations obtained from human cardiac fibroblasts, as previous reports were mainly devoted to rats [19–21]. The mechanism behind the influence of melatonin on collagen deposition is unknown, and the reason for this remains unclear. As membrane melatonin receptors appear to be absent from cardiac fibroblasts, it is possible that melatonin may act via retinoid nuclear receptors [22] or calmodulin, known as a cytoplasmatic receptor [23]. Thus, the negative effects of higher melatonin concentrations may be dependent on the downregulation of melatonin receptors.

Previous studies have found that melatonin administered at 30 or 60 µg/100 g of initial body weight increased the collagen level within a myocardial infarction scar. In addition, while pinealectomy countered the effect of melatonin injections, melatonin administration to pinealectomized rats reversed the effect of the pinealectomy: administration of 60 µg melatonin/100 g of initial body weight normalized collagen content [20]. This dose was assumed as physiological. While 0.1 µM melatonin also increased total collagen content in fibroblasts isolated from the myocardial infarction scar of rats, lower concentrations (0.01 µM–0.1 nM) were not effective [20].

Elsewhere, 50 µM to 100 µM concentrations of melatonin were also found to stimulate collagen synthesis in normal human bone cells (HOB-M) and human osteoclast cells (SV-HFO). Melatonin treatment increased the level of the carboxyterminal propeptide of procollagen type I, i.e., a marker of collagen synthesis, by 983% in HOB-M or 139% in SV-HFO [24].

Contradictory results are reported by experiments based on pharmacological doses of melatonin. These doses were reported to scavenge reactive oxygen radicals [25, 26]. Jiang and coworkers found pharmacological doses of melatonin administered orally (20 mg/kg) to have antifibrotic properties in a mouse heart perivascular fibrosis model caused by air pollution (PM25). Melatonin decreased oxidative injury and slowed the transformation of fibroblasts into myofibroblasts induced by PM25. Moreover, melatonin induced the SIRT3-dependent deacetylation of superoxide dismutase-2, thus increasing its reactive oxygen radicals scavenging potential. The authors propose that melatonin may be considered an antifibrotic compound protecting from air pollution-induced heart fibrosis [26].

Szeiffova and coworkers reported that pharmacological doses of melatonin (10 mg/kg/day) exerted antiarrhythmic and antifibrotic effects in spontaneously hypertensive rats or those treated with isoproterenol [25]. Melatonin administration (10 mg/kg/day) also demonstrated antifibrotic properties in the hearts of diabetic mice [27]. Hence, the final effect of melatonin appears to be dependent on the dose, and on the species of animal used for the experiment.

Our present findings also indicate that melatonin increases the level of mRNA for the procollagen type III α1 chain but not for the procollagen type I α1 chain. This supports previous data and suggests that treatment increases collagen type III synthesis. Hence, melatonin is supposed to exert its effect on collagen content by influencing collagen gene expression. However, the effect of the pineal hormone on the protein level of collagen type III expression is uncertain. This is the limitation of the study.

Melatonin did not alter the expression of procollagen type I or III genes in rat myocardial infarction scars, or fibroblasts isolated from them; such augmentation was only reported in myocardial infarction scars of pinealectomized rats [28]. Melatonin also reduced the mRNA and protein levels for collagen type I and III increased by air pollution (PM25) [26]. However, treatment did not influence MMP-2 release by cardiac fibroblast cultures, and pharmacological doses of melatonin did not influence MMP-2 activity within the hearts of spontaneously hypertensive rats or rats treated with isoproterenol [25].

Both surgical and pharmacological (induced by metoprolol) pinealectomies decreased collagen content within the myocardial infarction scar and retarded fibrotic processes in rats [20]. Evening application of metoprolol [inhibiting the night melatonin peak rise] decreased collagen content within the heart, but morning administration did not influence collagen content within the heart. This study suggested that the antifibrotic properties of metoprolol were dependent on the cessation of melatonin secretion during the dark phase in rats [20]. Moreover, metoprolol inhibited profibrotic cytokine secretion by epicardial adipose tissue in an obstructive sleep apnea canine model [29]. Fibrosis of the right ventricle was reduced by bucindolol in rats [30]. Overall, the discussed literature suggests that pharmacological blockade of the pineal gland may exert antifibrotic effects within the heart.

The present data reports that melatonin increases FGF-2 release but not cardiotrophin secretion. Augmented FGF-2 expression was noted in the hearts of spontaneously hypertensive rats, and this correlated with interstitial cardiac fibrosis [8]. Optimal concentrations of basic FGF support the proliferation of fibroblasts and both collagen type I and III secretion [30]; however, FGF-2 is also known to have antifibrotic properties [8]. Thus, the effect of FGF-2 on collagen deposition is not consistent, but its secretion is associated with fibrosis [8, 31].

These findings also indicate that melatonin increases both fibrosis and FGF-2 secretion. As melatonin appears to participate in the healing of myocardial infarction [20, 28] and it exerts profibrotic effects, we hypothesize that induction of FGF-2 synthesis alleviates melatonin-dependent collagen deposition and protects the heart from severe fibrosis development. The role of FGF-2 in regulating collagen deposition within the heart should be further investigated.

Melatonin was also found to decrease cardiac fibroblast cell count within the culture. This effect is accompanied by an elevation of necrotic cell counts and an acceleration of fibroblast proliferation. Thus, melatonin is supposed to induce two parallel processes, viz., cell elimination and proliferation, leading to the excessive replacement of cardiac fibroblasts. The decrease in the total cell count within the culture and low living cell count suggests that the net effect of melatonin treatment is cell death.

Our findings indicate that melatonin has a strong tendency to induce fibroblast necrosis; however, the obtained differences between groups are not statistically significant. Melatonin treatment increased the late apoptotic fibroblast count and tended to increase the numbers of both early apoptotic cells and propidium iodide-stained cells after 4 days of melatonin application. The pineal hormone induces endoplasmatic reticulum stress and increases gastric cancer cell apoptosis and autophagy [32]. In colorectal cancer, treatment induced apoptosis and inhibited cell proliferation, migration, and invasiveness [33], and increased cell death [34]. It also resulted in reduced cell viability and various cytotoxic effects in thyroid cancer cells [35]. Melatonin improved the proliferation and differentiation of PC12 cells cultured on polycaprolactone/gelatin composite fibrous electrospun scaffolds [36]. Moreover, melatonin may inhibit apoptosis and support endometrial stromal cell proliferation [37].

## Conclusion

Physiological concentrations of melatonin exert profibrotic effects within cardiac fibroblast cultures. Its action is dependent on the activation of procollagen type III expression: a marker of the early step of collagen synthesis. An increase in intracellular collagen additionally suggests that melatonin treatment increases collagen synthesis, but not collagen catabolism, dependent on MMP-2.

Melatonin-induced fibrosis is associated with decreased cardiac fibroblast count within the studied culture. Treatment resulted in both increased fibroblast proliferation and an insignificant increase in cell necrosis. It also stimulated FGF-2 secretion, which correlated with the fibrotic sites. Hence, melatonin appears to demonstrate pleiotropic effects. Our current findings, and previous data [19–21], indicate that melatonin may induce heart fibrosis; in addition, inhibiting its release would decrease the deposition of collagen within the heart. These speculations should be additionally confirmed in vivo*.* The main limitation of the present study is lack of the results from animal models or appropriate clinical observations (Table 1).

**Table 1:** Primers sequence

	Gene name	Primers sequence
1	*YWHAZ*	AAGTGCAATGGAGACCTTGG
		GTTGCCCTAGATGCAGAAGG
2	*RPLP13*	GGCACCATTGAAATCCTGAG GAAGGGGGAGATGTTGAGC

## Data Availability

The data are available from authors on reasonable request.
